# Clonal Exhaustion as a Mechanism to Protect Against Severe Immunopathology and Death from an Overwhelming CD8 T Cell Response

**DOI:** 10.3389/fimmu.2013.00475

**Published:** 2013-12-20

**Authors:** Markus Cornberg, Laurie L. Kenney, Alex T. Chen, Stephen N. Waggoner, Sung-Kwon Kim, Hans P. Dienes, Raymond M. Welsh, Liisa K. Selin

**Affiliations:** ^1^Department of Gastroenterology, Hepatology and Endocrinology, Hannover Medical School, Hannover, Germany; ^2^Program in Immunology and Virology, Department of Pathology, University of Massachusetts Medical School, Worcester, MA, USA; ^3^Clinical Institute of Pathology, Medical University of Vienna, Vienna, Austria

**Keywords:** clonal exhaustion, CD8, T cells, LCMV, immunopathology, lung, liver

## Abstract

The balance between protective immunity and immunopathology often determines the fate of the virus-infected host. How rapidly virus is cleared is a function of initial viral load, viral replication rate, and efficiency of the immune response. Here, we demonstrate, with three different inocula of lymphocytic choriomeningitis virus (LCMV), how the race between virus replication and T cell responses can result in different disease outcomes. A low dose of LCMV generated efficient CD8 T effector cells, which cleared the virus with minimal lung and liver pathology. A high dose of LCMV resulted in clonal exhaustion of T cell responses, viral persistence, and little immunopathology. An intermediate dose only partially exhausted the T cell responses and resulted in significant mortality, and the surviving mice developed viral persistence and massive immunopathology, including necrosis of the lungs and liver. This suggests that for non-cytopathic viruses like LCMV, hepatitis C virus, and hepatitis B virus, clonal exhaustion may be a protective mechanism preventing severe immunopathology and death.

## Introduction

Because of the property of viruses to infect cells, peptide cleavage products from many of their encoded proteins get incorporated into nascent class I major histocompatibility complex (MHC) molecules and get presented at the cell surface to CD8 T cells bearing T cell receptors (TCR) specific for the peptide-MHC complex. As a result, viral infections frequently stimulate very potent class I-restricted CD8 T cell responses capable of perforin- or FasL-dependent cytotoxicity, as well as IFNγ and TNFα production. Indeed, CD8 T cells are essential regulators of viral infection, playing important roles in the clearance of virus-infected cells and sometimes causing damaging immunopathology (1). The relative balance between protective immunity and immunopathology often determines the fate of the virus-infected host (2). A classic example is that of lymphocytic choriomeningitis virus (LCMV), where the same clone of T cells responsible for viral clearance can mediate a severe leptomeningitis if the virus is replicating in the brain (1, 3). The pathology that is induced by T cells during an acute infection most likely results from the inflammatory conditions brought about by the presence of high numbers of T cells lysing infected tissues via perforin and FasL, producing pro-inflammatory cytokines, including TNFα, and chemokines which recruit even more cells. Important factors in how rapidly virus is cleared include the initial viral load, rate of viral replication, and the efficiency of the activated antigen-specific T cells. Here, we demonstrate with three different inocula of LCMV clone 13 intravenously (i.v.) how the race between the virus and the T cell response can result in completely different outcomes. Mice given low doses of LCMV clone 13 developed a strong effector CD8 T cell response, which cleared the virus. If the viral load becomes high very rapidly it can result in clonal exhaustion of the T cell response and viral persistence (4–7) associated with little immunopathology, as was shown here in high dose LCMV clone13 infection. Here, we also demonstrate that if the mice were given an intermediate dose of LCMV clone 13, the immune response was able to develop and exhausted more slowly, leaving time for massive collateral damage and resulted in increased mortality with severe lung and liver necrosis.

## Materials and Methods

### Mice

C57BL/6 (B6, H-2^b^) and TCRβKO male mice were purchased from The Jackson Laboratory (Bar Harbor, ME, USA) and Taconic Farms (Germantown, NY, USA), respectively. Mice were used at 2–8 months of age. All mice were maintained under specific pathogen-free conditions in the University of Massachusetts Medical School, Department of Animal Medicine. All experiments were done in compliance with institutional guidelines as approved by the Institutional Animal Care and Use Committee at the University of Massachusetts Medical School.

### Viruses

Lymphocytic choriomeningitis virus (clone 13) is a RNA virus in the Old World Arenavirus family, and was propagated in BHK21 baby hamster kidney cells (8).

### Infection protocols

Mice were inoculated i.v. with low dose, 2 × 10^4^ pfu; medium dose, 2 × 10^5^ pfu; and high dose, 2 × 10^6^ pfu of LCMV clone 13. Control naïve mice were either left uninoculated or were inoculated with HBSS. The control mice were always age matched to the experimental group and housed exactly the same in pathogen-free conditions.

### Virus titration

Lymphocytic choriomeningitis virus titers in 10% tissue homogenate from spleens and serum were determined by plaque assays on ATCC vero cells, as described elsewhere (9).

### Peptides

Lymphocytic choriomeningitis virus epitopes NP_396_, D^b^ (FQPQNGQFI), GP_33_, D^b^ (KAVYNFATC), NP_205_ epitope (YTVKYPNL). Synthetic peptides were provided by Biosource International (Camarillo, CA, USA) or 21st Century Biochemicals (Marlboro, MA, USA) and used at a 90% level of purity.

### Cell surface and tetramer staining by flow cytometry

Single cell suspensions were prepared from splenocytes or peripheral blood. Erythrocytes were lysed with 0.84% NH_4_Cl solution. FACS staining was done as previously described in 96-well plates with fluorochrome-labeled mAbs, anti-CD8 (clone 53-6.7, BD Pharmingen), anti-CD44 (clone IM7), and PD-1 (clone J43). Tetramer staining was done as previously described using phycoerythrin (PE) and/or allophycocyanin (APC) labeled tetramers (10). Samples were analyzed with a Becton Dickinson FACSCalibur flow cytometer (San Jose, CA, USA) or a LSRII (San Jose, CA, USA) and FlowJo software (Tree Star, Inc., Ashland, OR, USA). All surface mAbs were purchased from BD Pharmingen, San Diego, CA, USA or eBioscience, San Diego, CA, USA. MHC class I peptide tetramers specific for LCMV-NP_205_/K^b^, LCMV-NP_396_/D^b^, and LCMV-GP_33_/D^b^ were prepared as previously described (10).

### Intracellular cytokine staining

Cells (10^6^) were stimulated either with medium, or 1–5 μM synthetic peptide as previously described (10). Intracellular cytokine-producing cells were detected with anti-IFNγ (clone XMG1.2), anti-TNFα (clone MP6-XT22), and anti-IL-2 (clone JES6-5H4) mAbs. IgG-isotype mAbs were used in the same assay. The mAbs were purchased from BD Pharmingen, San Diego, CA, USA or eBioscience, San Diego, CA, USA. The samples were analyzed as described above.

### CD8 depletion

Mice were injected i.p. on day-1 and -7 of LCMV clone 13 infection with 100 μg of CD8 (clone 2.43) antibody.

### Histology

At day 14 after LCMV challenge, lungs and livers from LCMV clone 13-infected mice were collected, fixed in 10% neutral buffered formaldehyde and paraffin-embedded. Tissue sections (5 μm) were stained with hematoxylin and eosin and analyzed microscopically by a pathologist. Scoring of lung pathology was graded based on a scale outlined below and based on previously published studies (11–13). Scoring of lung pathology was graded by one pathologist, who was blinded in regards to treatments the mice had received.

1. Mild interstitial mononuclear infiltrates, disorganized BALT, perivascular edema.
2. Moderate interstitial mononuclear infiltrates, small amount of organized BALT, pulmonary edema.
3. Moderate interstitial mononuclear infiltrates, pulmonary edema, enhanced organized BALT, mild consolidation.
4. Severe interstitial mononuclear infiltrates, greatly enhanced pulmonary edema, enhanced organized BALT, moderate consolidation and moderate necrotizing bronchiolitis.
5. Severe interstitial mononuclear infiltrates, greatly enhanced pulmonary edema, enhanced organized BALT, severe consolidation, severe necrotizing bronchiolitis and vasculitis involving more than half of the lung.

The scoring on each individual mouse was done on four to five different sections of the lung representing four to five different lobes of the whole lung assessing both qualitative and quantitative changes in histology. The histology photographs showing high power views of a small portion of one lobe of the lung demonstrate examples of the types of pathology observed in different treatment groups, but evaluations of the whole lung were used for scoring.

### ALT assay

Serum alanine aminotransferase (ALT) levels were determined using an ALT UV-kinetic method reagent set (D-Tek, Bensalem, PA, USA). Fifteen microliters of serum was mixed with 150 μL of substrate and absorbance at was read at 340 nm. ALT concentration was calculated as the average change in absorbance/min multiplied by 1768. ALT (IU/L) is equal to the change in absorbance/min multiplied by total reaction volume (0.165 mL) *1000 to convert IU/mL to IU/L. This is divided by the millimolar absorptivity of NADH (6.22) multiplied by the sample volume (0.015 mL) multiplied by the light path (1.0 cm). This equation simplifies into the change in absorbance/min multiplied by 1768.

### Statistical analyses

The one-way ANOVA test with a Bonferroni post-test was used when there were more than two groups. The Student’s *t*-test was used as indicated in the figures when only comparing two groups. The Mantel–Cox Mortality test was used for mortality studies.

## Results

### Increased immunopathology and mortality during LCMV medium dose infection

**Figure 1:**
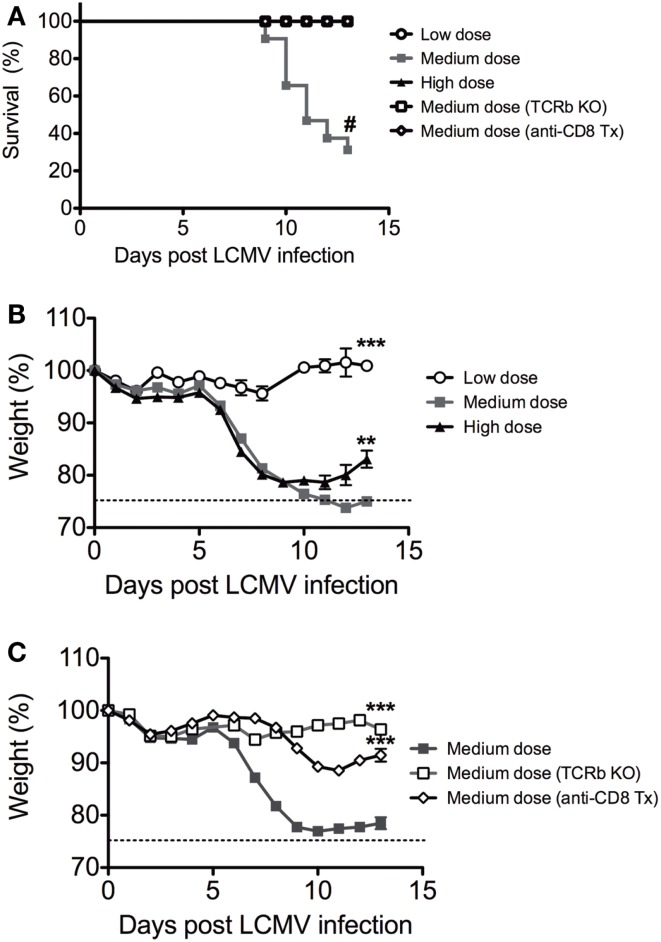
**Balance between viral load and CD8 T cell response determines disease outcome to LCMV clone 13 infection**. **(A)** Increased mortality in C57BL/6 mice infected with medium dose of LCMV clone 13 (2 × 10^5^ pfu) i.v. as compared to low dose (2 × 10^4^ pfu) or high dose (2 × 10^6^ pfu) depleted of CD8 T cells with anti-CD8 mAb or TCRβ KO mice were protected from lethal effect of medium dose (^#^*p* = 0.05; *n* = 11–33 mice/group) (Mantel–Cox Mortality, ^#^*p* < 0.002). The symbols, which cannot be discerned are all overlaid on the 100% line. **(B)** Increased weight loss in both medium dose and high dose LCMV clone 13-infected mice, but high dose began to regain weight at day 13 while medium dose do not (medium vs. high, **p = 0.008; medium vs. low, ***p = 0.0002; *n* = 11–12 mice/group). **(C)** Mice depleted of CD8 T cells with anti-CD8 mAb or TCRβ KO mice were protected from weight loss during medium dose (medium vs. TCRβ KO, ***p = 0.0001; medium vs. CD8 depleted, ***p = 0.0002; TCRβ KO vs. CD8 depleted ***p = 0.003, *n* = 9–22 mice/group).

We infected C57BL/6 mice intravenously with low dose (2 × 10^4^ pfu), medium dose (2 × 10^5^ pfu), and high dose (2 × 10^6^ pfu) LCMV clone 13 i.v. There was significantly higher mortality in the medium dose group, with only 25% of mice surviving at Day 13 (Figure 1A). Both the medium and high dose groups experienced a rapid and significant >20% weight loss beginning at day 6 after infection, with the high dose group starting to recover after day 10 while the medium dose group did not recover even by day 13 (Figures 1B,C). All of the mice that survived the medium dose infection were persistently infected with LCMV, similar to the high dose group. The dramatic immunopathology was mediated by the T cell response, as demonstrated by the fact that TCRβ KO mice infected with the medium dose did not have any significant weight loss or death, in contrast to WT controls (Figures 1A,C). The TCRβ KO mice had a slightly higher viral load than the WT controls at day 13 post infection and yet did not have pathology (log10 pfu/spleen: B6 5.7 ± 0.13 SEM; TCRβ KO 6.3 ± 0.13; *n* = 5/group; *p* < 0.02). The CD8 T cells were the major T cell population involved in mediating this effect, as demonstrated by depletion of CD8 T cells with mAb. B6 mice depleted of CD8 T cells prior to infection with the medium dose of LCMV also had significantly less weight loss and decreased mortality than the WT controls (Figure 1C) without a significant difference in viral load in the spleen at day 13 post infection (log10 pfu/spleen: B6 4.9 ± 0.5 SEM; anti-CD8 tx 5.3 ± 0.07 *n* = 5–9/group).

### Partial clonal exhaustion in the medium dose-infected mice

**Figure 2:**
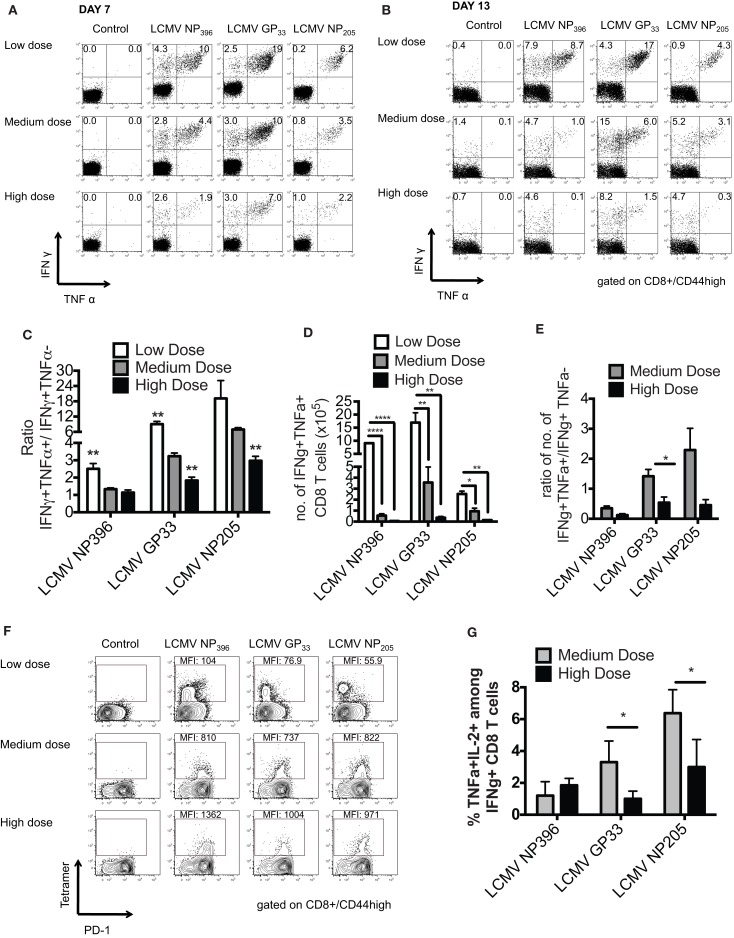
**Partial clonal exhaustion in the LCMV clone 13 medium dose-infected mice**. The functionality of the epitope-specific CD8 T cells in the three groups of mice was determined by ICS staining for the presence of IFNγ and/or TNFα-producing cells and calculating the ratio of IFNγ+TNFα+/IFNγ+ cells at days 7 **(A)** and 13 **(B)** post infection in peripheral blood **(A)** and spleen **(B)**. The high dose clone 13-infected mice had significant exhaustion of NP396-, GP33-, and NP205-specific responses as defined by loss of double producing INFγ/TNFα cells by day 13 **(C)**. The medium dose mice had evidence of only partial exhaustion of their CD8 T cell response in this same time frame. They had greater frequency of IFNγ/TNFα producing cells by all three CD8 epitope-specific responses on day 7 and day 13 than high dose mice, although significantly less than the low dose group (NP396: low vs. medium, high vs. low; GP33: low vs. medium, medium vs. high, high vs. low; NP205: medium vs. high, high vs. low, **p < 0.01; *n* = 5–7 mice/group). **(D)** The medium dose mice also had increased total numbers of GP33-specific IFNγ+TNFα+ CD8 T cells in the spleen at day 13–14 post infection (*n* = 3–6 mice/group pooled from three experiments). Data analyzed by a one-way ANOVA and Tukey test. ****p < 0.0001, **p < 0.01, *p < 0.05. **(E)** These medium dose mice also demonstrated an increased ratio in IFNγ+TNFα+/IFNγ+ producing cells in the total numbers of GP33-specific cells in the spleen at day 13–14 post infection than high dose mice. **(F)** CD8 expression of cell surface marker PD-1 on epitope-specific cells at day 10 post infection was intermediate in the medium dose as compared to the high and low dose groups, consistent with partial exhaustion. This is representative of two similar experiments. **(G)** There was also a significantly greater frequency of LCMV-GP33 and NP205-specific CD8 T cells producing IL-2 and TNFα on day 11 post infection in the medium dose as compared to the high dose-infected mice (*p < 0.05; *n* = 4 mice/group). These data are representative of two similar experiments.

Persistent infection leads to a disruption of the normal immunodominance hierarchy and function of CD8 T cell responses during high dose LCMV clone 13 infection referred to as clonal exhaustion (6, 14). Clonal exhaustion occurs by a stepwise loss of function due to high antigen load and loss of CD4-help. Cells first lose cytotoxic abilities, then IL-2 production followed by the ability to produce TNFα and finally IFNγ. The final stage of exhaustion is the deletion of antigen-specific cells by apoptosis making exhaustion both a qualitative and quantitative. CD8 T cell functional impairment occurs in a hierarchical fashion in chronically infected mice. Production of IL-2 and the ability to lyse target cells *in vitro* are the first functions compromised, followed by the ability to make TNFα, while IFNγ production is most resistant to functional exhaustion. Antigen appears to be the driving force for this loss of function, since a strong correlation exists between the viral load and the level of exhaustion (15). Epitopes presented at higher levels *in vivo* result in physical deletion, such as NP396, while those presented at lower levels, such as GP33 and NP205, induce functional exhaustion. These published data would suggest that antigen levels drive the hierarchical loss of different CD8 T cell effector functions during chronic infection, leading to distinct stages of functional impairment and eventually to physical deletion of virus-specific T cells. Thus, we determined the functionality of the epitope-specific CD8 T cells in the three groups of mice by intracellular cytokine staining (ICS) for the presence of IFNγ- and/or TNFα-producing cells and calculating the ratio of IFNγ+TNFα+/IFNγ+ cells at days 7 and 13 post infection. The high dose clone 13-infected mice had significant exhaustion of NP396-, GP33-, and NP205-specific responses, as defined by loss of the double positive IFNγ/TNFα producing cells by day 13 (Figures 2A–E). The medium dose mice had evidence of only partial exhaustion of their CD8 T cell response in this same time frame. They had significantly more of the double positive IFNγ/TNFα producing T cells by all three epitope-specific CD8 T cell responses on day 7 and day 13 than did high dose-infected mice, although significantly less than the low dose group. Expression of the prototypic cell surface marker of exhaustion, PD-1 (14, 16) on epitope-specific CD8 T cells at day 10 post infection was intermediate in the medium dose as compared to the high and low dose groups, consistent with partial exhaustion (Figure 2F). There was also a significantly greater portion of the functional LCMV-GP33 and -NP205-specific CD8 T cells in the medium dose mice producing three cytokines including IL-2 (Figure 2G). These results would suggest that at the medium dose of virus the viral load is high enough to result in a partial exhaustion phenotype (6) and these sub-optimally functioning CD8 T cells are able to mediate the induction of severe immunopathology leading to death.

### Increased lung and liver pathology in medium dose-infected mice

**Figure 3:**
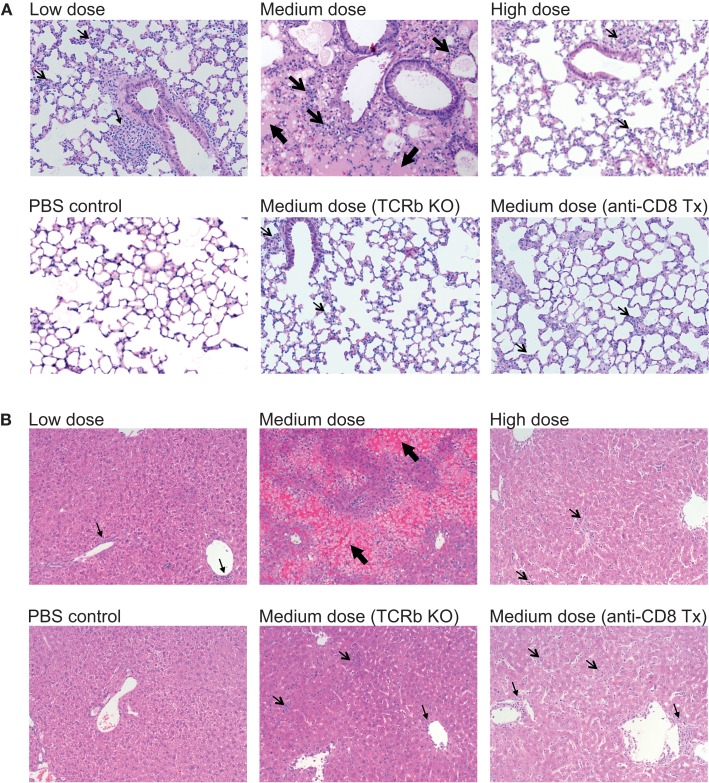
**Enhanced immunopathology in the lung and liver during medium dose LCMV clone 13 infection**. **(A)** Low dose LCMV clone 13 infection i.v. induces mild interstitial infiltrates as demonstrated in histology (H&E stain) sections of the lung at day 13. Medium dose LCMV clone 13 infection i.v. induces severe pulmonary edema, interstitial infiltrates with consolidation, enhanced bronchus associated tissue (BALT), and necrotizing bronchiolitis. High dose LCMV clone 13 infection i.v. induced minimal pathology with few interstitial infiltrates. Mice depleted of CD8 T cells with anti-CD8 mAb or TCRβ KO mice were protected from severe lung pathology having only minimal interstitial infiltrates. **(B)** Histological sections of the liver (H&E stain) showed serious bridging necrosis in the medium dose LCMV clone 13-infected mice at day 13, while the high and low dose mice as well as the TCRβ KO and CD8 depleted medium dose mice had only periportal and sinusoidal inflammatory infiltrates. Arrows indicate the areas of significant pathology.

**Figure 4:**
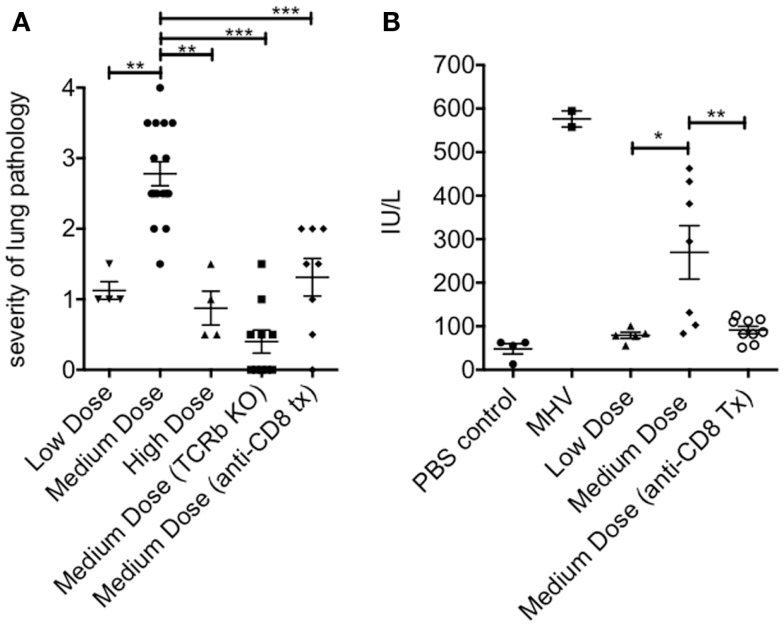
**Increased pathology in the lung and liver during medium dose LCMV clone 13 infection**. **(A)** The severity of the lung pathology was scored using our established histology scale (see Materials and Methods). The lungs of mice infected with LCMV clone 13 medium dose i.v. had significantly more severe lung pathology than low dose or high dose-infected mice at day 13 post infection. This pathology was abrogated by anti-CD8 mAb treatment or by infecting TCRβ KO mice (**p = 0.003, ***p < 0.001; *n* = 4–9 mice/group) **(B)** Severity of liver pathology was assessed by measuring the levels of the liver enzyme ALT at day 13 post infection (see Materials and Methods). ALT levels were significantly increased in medium dose-infected mice as compared to low dose. Treatment with anti-CD8 at the time of infection with medium dose prevented this increase in ALT levels. Positive control for this ALT assay is day 13 mice infected with mouse hepatitis virus (MHV) (*N* = 2) (*p = 0.02, **p = 0.008; *n* = 6–10 mice/group). These data represent two separate experiments pooled.

The lung immunopathology in the surviving medium dose mice as assessed by histology demonstrated severe pulmonary edema and interstitial infiltrates with consolidation, enhanced bronchus associated tissue (BALT), and necrotizing bronchiolitis (Figure 3A). Using our established method of scoring lung pathology (11–13) the medium dose mice had significantly more pathology than either the high and low dose mice or the TCRβ KO and the CD8 T cell-depleted mice, all of which had minimal interstitial infiltrates, consistent with this being CD8 T cell-mediated pathology (Figure 4A).

The livers of the medium dose mice also had significantly increased liver pathology (Figures 3B and 4B) as demonstrated by increased liver enzyme, alanine aminotransferase (ALT), in their serum as compared to low dose, TCRβ KO, and CD8 T cell-depleted mice. Histological sections of the liver showed serious bridging necrosis in the medium dose mice (Figure 3B), while the high and low dose mice as well as the TCRβ KO and CD8 depleted medium dose mice had only periportal and sinusoidal inflammatory infiltrates. We also examined the lymph nodes of these mice and found that with all three doses of LCMV clone 13 they essentially maintained their normal architecture meaning that there was recruitment of lymphocytes in T cell zones and increased numbers of germinal centers with infection but there was no overall destruction or necrosis of the lymphoid structures as has been reported for high dose LCMV-WE infection (17, 18) (data not shown).

## Discussion

**Figure 5:**
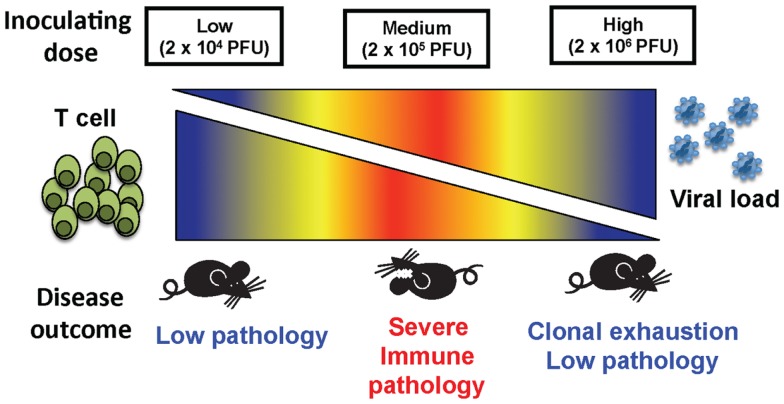
**The balance between viral load and T cell response determined the disease outcome of mice inoculated at three different doses of virus**. A low dose of LCMV results in a strong T cell response that quickly clears virus and causes little immunopathology. At a high dose of LCMV, the T cell response is clonally exhausted leading to limited immunopathology even though there is persistent virus. An intermediate dose of LCMV induces only a partial clonal exhaustion and mice develop severe immunopathology with up to 75% mortality.

These results demonstrate that, depending on the initial dose of non-cytopathic viruses such as LCMV clone 13, a rapidly replicating virus, disease outcome can differ greatly (Figure 5). The fact that high dose LCMV clone 13 infection resulted in clonal exhaustion and viral persistence has been previously reported, and is a well-established model for viral persistence (4–7, 19). As shown here, the limited pathology occurring after high dose infection suggests that extensive clonal exhaustion may be a beneficial immune mechanism that prevents death from an overwhelming CD8 T cell response. The importance of this extensive clonal exhaustion was demonstrated by using the medium dose of LCMV. At medium dose LCMV clone 13, the CD8 T cell response was only partially exhausted, leaving a suboptimal response that was unable to clear the virus yet persisted at a level of function that caused severe immune pathology that could kill the host. The medium dose mice which survived were finally able to exhaust their immune response and became persistently infected, much like the high dose-infected mice. Persistent LCMV clone 13 infection is ultimately cleared in B6 mice by day 60, and epitope-specific CD8 T cells return, except for the high affinity NP396 T cells, which are eliminated by apoptosis (4, 14, 20) in the presence of functional LCMV-specific CD4 T cells (21, 22).

High dose clonal exhaustion has been extensively studied (4–7, 14, 17, 22, 23) and it has been suggested to be a mechanism for the virus to survive by not killing its host. However, we would suggest it is also a mechanism of the host to survive high doses of pathogens whether it is from a very high initial inoculum as may be received in a direct intravenous infection or from pathogens that replicate extremely fast (15). For instance, Hepatitis B virus (HBV) infection can present with a spectrum of disease from an acute resolving infection to a chronic persistent infection or a very fulminant hepatitis with high levels of T cells and a sudden and severe liver destruction and failure (24). In fact, PD-1 expression on virus-specific CD8 T cells has been correlated with the outcome of HBV (25). Delayed PD-1 expression on HBV-specific CD8 T cells was associated with delayed exhaustion of the CD8 T cell response and subsequent acute liver failure. It is possible that these three different outcomes can be partially explained by the fine balance between the HBV load and the efficiency of each individual’s CD8 T cell response. Other earlier studies have suggested that there may be a fine balance between viral dose and the qualitative and quantitative characteristics the CD8 T cell response, which impacts disease outcome either by infecting genetically modified mice so that T cell receptor signaling is attenuated (26), or using mathematical models (27), or transferring in different quantities of antigen-specific cells prior to challenge with extremely high doses of virus (28).

The data presented here are also actually the basis for our observation that NK cells play a role in controlling T cell exhaustion. Here, we make the point that T cell exhaustion is a positive event, which prevents mortality and severe immunopathology. However, we do not cover all the potential mechanisms which contribute to the induction of this phenomenon. Our data suggests that the key inducer of pathology is activated CD8 T cells. However, other immune cells also contribute by controlling CD8 T cell activation (like CD4 cells and NK cells). In fact, we recently found that NK cells act as a rheostat and play a role in regulating CD8 T cell exhaustion (29). NK cells can kill activated CD4 T cells, resulting in the loss of CD4-help and exhaustion of CD8 T cells. Another study found that using different doses of LCMV clone 13 could induce different levels of weight loss with an intermediate dose causing the severest weight loss. They also suggested that CD4 cells play a role in this phenomenon as they showed that ablation of the CD4 T cell response by depletion reverted this phenotype (30).

Our studies could lead to a counter intuitive treatment strategy for other lethal arenaviruses, such as Lassa fever. Perhaps these patients should be treated short-term with anti-CD3 (31, 32) or anti-CD8 as their immunopathology and death may be T cell-mediated and preventable. Generalized immunosuppressive therapy such as corticosteroids may have been tried in the past, but these treatments would lead to suppression of all aspects of the immune response, leaving the host helpless. Just focusing on the CD8 T cell responses or even particular CD8 functions may improve outcome without disabling the complete immune system.

## Conflict of Interest Statement

The authors declare that the research was conducted in the absence of any commercial or financial relationships that could be construed as a potential conflict of interest.

## Abbreviations

ICS, intracellular cytokine staining; IFN, interferon; LCMV, lymphocytic choriomeningitis virus; TCR, T cell receptor; TNF, tumor necrosis factor.
